# Comprehensive serum glycopeptide spectrum analysis with machine learning for non-invasive early detection of gastrointestinal cancers

**DOI:** 10.1016/j.csbj.2025.10.067

**Published:** 2025-11-01

**Authors:** Yuichi Hisamatsu, Kazuhiro Tanabe, Kensuke Kudo, Hirofumi Hasuda, Eiji Kusumoto, Hideo Uehara, Rintaro Yoshida, Mitsuhiko Ota, Yoshihisa Sakaguchi, Chihiro Hayashi, Mikio Mikami, Tetsuya Kusumoto

**Affiliations:** a Department of Gastroenterological Surgery and Clinical Research Institute Cancer Research Division, National Hospital Organisation Kyushu Medical Center, Fukuoka, Japan; b Medical Solution Promotion Department, Medical Solution Segment, LSI Medience Corporation, Itabashi-ku, Tokyo, Japan; c Department of Obstetrics and Gynecology, Tokai University School of Medicine, Isehara, Kanagawa, Japan

**Keywords:** Gastrointestinal cancer, Comprehensive serum glycopeptide spectra analysis, Neural network, Glycopeptide, Mass spectrometry, Glycomics

## Abstract

**Purpose:**

Gastrointestinal cancers, including colorectal cancer (CRC), gastric cancer (GC), and esophageal cancer (EC), are among the most common and lethal malignancies worldwide. Early detection is critical for improving patient outcomes, but the current diagnostic methods, such as endoscopy, are burdensome, costly, and inaccessible for widespread screening. Here, we have identified the transformative potential of non-invasive blood-based diagnostics by integrating advanced glycan biomarkers and machine learning.

**Experimental design:**

This study analyzed serum samples from 296 CRC, 180 GC, and 42 EC patients, alongside 590 healthy controls. Nine conventional tumor markers were quantified and 1688 enriched glycopeptides (EGPs) were identified via liquid chromatography-mass spectrometry. Using Comprehensive Serum Glycopeptide Spectrum Analysis (CSGSA), EGPs were integrated with conventional markers into machine learning models, including neural networks, to develop and validate diagnostic frameworks.

**Results:**

Two glycopeptides, α1-antitrypsin at Asn271 and α2-macroglobulin at Asn70, were identified as highly cancer-specific biomarkers. Integrating these glycopeptides, tumor markers, and EGPs significantly improved the diagnostic performance. The neural network-based model achieved area under the curve values of 0.966, 0.992, and 0.995 for CRC, GC, and EC, respectively, with respective positive predictive values of 54.5 %, 35.3 %, and 11.1 %, exceeding non-invasive diagnostic benchmarks. Remarkably, the CSGSA approach differentiated cancer types with high accuracy, even in early-stage disease.

**Conclusion:**

CSGSA represents a breakthrough in non-invasive gastrointestinal cancer diagnostics, combining glycopeptide profiling with machine learning to achieve unprecedented accuracy. This method provides a cost-effective and scalable alternative to invasive procedures and may have potential utility in general health screening, which warrants further investigation.

## Graphical Abstract

**Figure:**
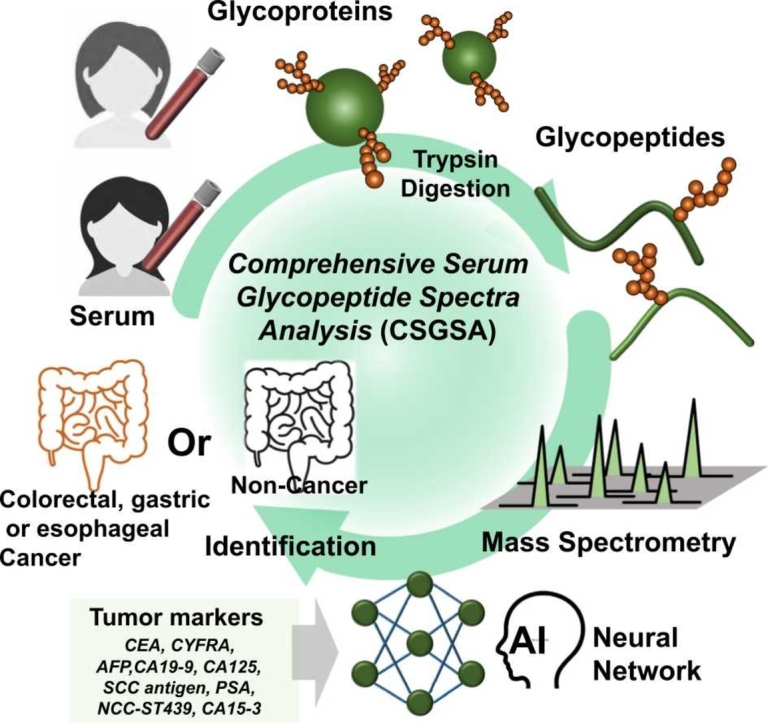

## 1 Introduction

Gastrointestinal cancers, including colorectal cancer (CRC), gastric cancer (GC), and esophageal cancer (EC), are among the most prevalent and fatal cancers worldwide. While early diagnosis can significantly improve patient survival rates, these cancers are often detected at advanced stages because of the lack of symptoms in the initial stages [1], [2]. Although endoscopic examination, a conventional diagnostic method, is effective for early cancer detection, the significant physical burden, high cost, and requirement for specialized skills present challenges for patients. Non-invasive and cost-effective diagnostic methods, such as blood-based biomarker testing, have garnered attention. However, diagnosis relying on a single biomarker often lacks sufficient sensitivity and specificity for early cancer detection [3], [4], [5], [6]. Consequently, approaches combining multiple biomarkers have shown promise, with multi-marker diagnostic methods using machine learning techniques potentially providing substantial improvements in diagnostic accuracy [7], [8].

Early and accurate detection of gastrointestinal cancers remains a critical unmet clinical need, as current diagnostic methods are often invasive, costly, and unsuitable for large-scale screening. In this study, we aimed to develop a practical and scalable non-invasive diagnostic approach by leveraging serum glycopeptide profiling combined with machine learning, placing particular emphasis on practicality and feasibility. It was essential that the obtained results be reproducible and independent of variations in pretreatment conditions or equipment settings. We developed a comprehensive diagnostic model as a novel diagnostic approach using methods established through foundational research, focusing on serum glycopeptides in combination with conventional tumor markers [9], [10], [11]. This model aims to enable the early detection of gastrointestinal cancers through detailed enriched glycopeptide (EGP) profiling, integrated with a machine learning-based Comprehensive Serum Glycopeptide Spectra Analysis (CSGSA) approach. Compared with conventional blood tests, this model can potentially achieve a higher diagnostic accuracy and thereby contribute to the early detection of cancer.

## 2 Materials and methods

### 2.1 Study design and patient cohort

This was a retrospective, observational, case-control study. All patients and healthy volunteers who visited the hospital between July 2018 and March 2021 were eligible for inclusion. Random sampling and blinding were not implemented. Under a framework assuming an alpha error of 0.05 and beta error of 0.2, considering that the average expression levels of the target markers would differ by approximately half of the standard deviations between the cancer and healthy groups, the minimum sample size was estimated to be approximately 100. We enrolled 296 CRC patients, 180 GC patients, and 42 EC patients, as well as 590 healthy volunteers (Table 1). Serum samples were collected from patients at the time of cancer detection and before any treatment or surgery. Japanese patient serum samples were obtained from National Hospital Organization Kyushu Medical Center (Fukuoka, Japan). Caucasian cancer patient serum samples were obtained from KAC Corporation (Kyoto, Japan) and Sanfco Ltd. (Tokyo, Japan). Serum samples of Japanese healthy volunteers were obtained from LSI Medience Corporation (Tokyo, Japan) and SOIKEN (Osaka, Japan), while those of Caucasian, African American, and Hispanic healthy volunteers were obtained from KAC Corporation and Sanfco Ltd. The Institutional Review Boards of National Hospital Organization Kyushu Medical Center (IRB registration number: 17C299; December 20, 2017) and LSI Medience Corporation (IRB registration number: MS/Shimura 17–19; January 22, 2018) approved the use of patient clinical information and serum samples. All research methods and procedures were conducted strictly according to the ethical principles outlined in the Declaration of Helsinki, as well as in full compliance with relevant institutional, national, and international guidelines. We obtained written informed consent from all patients and volunteers. The specific inclusion criteria for this study were as follows: (i) patients diagnosed with primary cancer via imaging or histological analysis; (ii) patients at initial diagnosis or experiencing a recurrence of cancer who had not yet commenced treatment; and (iii) patients aged 20 years or older at the time of consent. The exclusion criteria included: (i) patients with severe renal, hepatic, respiratory, or cardiac dysfunction, or concurrent infectious diseases; (ii) patients deemed unsuitable for study enrollment by the attending physician; and (iii) patients who had already started any form of treatment. Cancer staging was performed according to the TNM classification system provided by the Union for International Cancer Control (UICC) [12]. Blood samples were collected via venous puncture before surgery or treatment. The sera were separated from blood cells through centrifugation at 2000 ×g for 10 min at room temperature within 8 h of collection and stored at −80°C until analysis. All samples were only analyzed once because we had previously validated their accuracy and reproducibility [13].

**Table 1:** Demographic characteristics of the patients.

Condition	Age	Number	Sex (Man Ratio)	Stage	Race
Healthy Volunteers (HE)	48.2 (±12.2)	590	50.3 %		Asian (297) Caucasian (114) African American (115) Hispanic (63) Mixed Ethnicities (1)
Colorectal Cancer (CRC)	69.9 (±11.1)	296	50.7 %	Stage I (76) Stage II (96) Stage III (79) Stage IV (38) Unclassified (7)	Asian (276) Caucasian (20)
Gastric Cancer (GC)	69.8 (±10.8)	180	67.8 %	Stage I (73) Stage II (47) Stage III (39) Stage IV (21)	Asian (160) Caucasian (20)
Esophageal Cancer (EC)	61.3 (±9.8)	42	73.8 %	Stage I (5) Stage II (18) Stage III (15) Stage IV (4)	Asian (18) Caucasian (24)
Total	58.0 (±15.7)	1108	54.2 %		

The numbers in the parentheses indicate the standard deviation of the age or number of participants.

### 2.2 Tumor marker analyses

Nine tumor markers, carcinoembryonic antigen (CEA), carbohydrate antigen 19–9 (CA19–9), cytokeratin 19 fragment (CYFRA), NCC-ST-439, cancer antigen 125 (CA125), prostate specific antigen (PSA), cancer antigen 15–3 (CA15–3), alpha-fetoprotein (AFP), and squamous cell carcinoma antigen (SCCA), were all analyzed in serum samples by LSI Medience Corporation, a clinical testing laboratory (Tokyo, Japan).

### 2.3 Sample preparation and liquid chromatography-tandem mass spectrometry (LC-MS/MS)

Our glycopeptide analysis employs two distinct strategies. The first approach focuses on identifying novel cancer markers from among over 10,000 EGPs. The second approach involves using these EGPs to develop a machine learning model capable of identifying cancers. The second method, known as CSGSA, analyzes more than 1000 EGPs, integrating conventional tumor markers by machine learning. CSGSA is anticipated to significantly reduce the rates of both false positives and false negatives by combining clinically validated tumor markers, which have limited sensitivity, with glycan chains that are highly responsive to cancer onset. The sample preparation and analysis methods were adapted from those outlined in our previous work [11]. Modifications included the following steps: A 20 μL aliquot of serum was mixed with 120 μL of acetone containing 10 % trichloroacetic acid to precipitate proteins. These proteins were subsequently denatured using a buffer composed of 80 μg urea, 100 μL Tris-HCl buffer (pH 8.5), 10 μL of 0.1 M EDTA, 5 μL of 1 M Tris (2-carboxyethyl) phosphine hydrochloride, 38 μL water, and 40 μL of 1 M 2-iodoacetamide. The mixture was then transferred to a 30 kDa ultrafiltration tube (Amicon Ultra 0.5 mL, Millipore Corp., MA, USA) to eliminate the denaturing agents. Protein digestion was performed on the filter using 200 μL of 0.1 M Tris-HCl buffer (pH 8.5), 20 μL of 0.1 μg/μL trypsin (Wako Pure Chemical Industries, Osaka, Japan), and 20 μL of 0.1 μg/μL lysyl endopeptidase (Fujifilm Wako Pure Chemical Industries) and incubated for 16 h at 37°C. After the digestion process, the mixture was centrifuged at 11,500 ×g for 30 min. The resulting filtrate, which contained both digested peptides and glycopeptides, was then transferred to a 10 kDa ultrafiltration tube (Amicon Ultra 0.5 mL, Millipore Corp.) to separate glycopeptides from non-glycosylated peptides [14]. The trapped compounds following 10 kDa ultrafiltration, referred to as the EGPs, were subsequently analyzed using a liquid chromatography quadrupole time-of-flight mass spectrometer (HP1200 + 6520, Agilent Technologies, Palo Alto, CA, USA), equipped with a C18 column (Inertsil ODS-4, 2 μm, 100 Å, 100 mm × 2.1 mm ID, GL Science, Tokyo, Japan). The EGPs were eluted using a gradient program at a flow rate of 0.2 mL/min and temperature of 40°C: starting with 15–30 % mobile phase B for the first 7 min, increasing to 30–50 % mobile phase B from 7 to 12 min, followed by a 2-minute hold at 100 % mobile phase B. The mobile phase A consisted of 0.1 % formic acid in water, while mobile phase B was composed of 0.1 % formic acid in 9.9 % water and 90 % acetonitrile. The mass spectrometer was operated in the negative ion mode with a capillary voltage of 4000 V. All samples were analyzed only once. In total, 1688 EGPs were chosen from more than 30,000 detected peaks by following three steps: (i) removing low reproducibility peaks (coefficient of variation (CV) > 50 %), (ii) removing low reliability peaks (signal to noise < 5), and (iii) removing isotope, adduct, and fragment ions. Subsequently, residual 1688 EGP peaks were used for biomarker screening and CSGSA diagnostics. The analytical reproducibility of the 1688 EGP peaks is shown in Supplementary Figure 1.

### 2.4 Data processing

The methods for data processing are discussed in detail in previous publications [14], [15]. In summary, the LC-MS raw data were exported to CSV format using Mass Hunter Export software (Agilent Technologies). Using R software (R 3.2.2, R Foundation), we extracted the peak positions (retention times and *m/z* values) and peak areas. Marker Analysis software, provided by LSI Medience Corporation (Tokyo, Japan), was then employed to align all peak areas, minimize noise, and correct any discrepancies [14]. The tolerances for *m/z* and retention time during peak alignment and assignment were maintained at 0.06 Da and 0.3 min, respectively. For each sample, the relative levels of 1688 EGPs were determined by calculating the ratios relative to a quality control standard. The sample was randomly split into two sets: 70 % for training and 30 % for testing. A model was then trained using the training set, with its accuracy subsequently evaluated on the test set. Two models were evaluated: XGBoost and neural network (NN). This process was iterated 10 times to ensure the robustness of the model. The results from each iteration were aggregated and receiver operating characteristic (ROC) curve analysis was conducted. Area under the ROC curve (AUC) values were then calculated from these cumulative results. The model outputs predicted values ranging from zero to one. To improve visualization and cutoff definition, these values were transformed into CSGSA scores using the following formula:

CSGSA score = -log_10_ (1 − predicted value)

This transformation expands the intermediate range of values that would otherwise cluster near 0 or 1, thereby facilitating clearer histograms and cutoff identification. To avoid divergence near 1, scores above 10 were capped at 10. Importantly, this transformation does not alter ROC-AUC or the underlying predicted probabilities, which remain available for direct interpretation.

### 2.5 Identification of the glycopeptides contributing to gastrointestinal cancer discrimination

To identify glycopeptide structure, we assessed the retention times, single mass spectra, and tandem mass spectrometry (MS/MS) patterns of target glycopeptides against those derived from commercially available purified human serum proteins. These proteins included alpha-1-acid glycoprotein, complement C8, complement C9, complement factor H, fibrinogen, haptoglobin, α2-macroglobulin, α1-antitrypsin, and transferrin, all sourced from Sigma-Aldrich (St. Louis, MO, USA). Following digestion, these glycopeptides from patients with gastrointestinal cancers were analyzed together. Matching retention times, mass spectra, and MS/MS patterns between the patient-derived glycopeptides and standards allowed us to identify the source of the glycopeptides. Considering all possible glycan structures and peptide sequences to which glycans could bind, all possible glycopeptide molecular weights were calculated and compared with the glycopeptide molecular ion peak actually detected. A structure of the glycopeptide was confirmed when its theoretical molecular weight matched the observed value within a 0.03 Da tolerance. For selected EGPs such as AT271-FSG and MG70-FSG, we annotated the glycosylation site and glycan composition, demonstrating that specific glycosylation sites contributed significantly to the observed diagnostic performance.

### 2.6 Statistical analysis

We developed the machine learning model using Python (version 3.12, 64-bit). To compare the EGP levels between gastrointestinal cancer and healthy control samples, we employed the Student's *t*-test, assuming a parametric distribution for all EGPs. Missing data, primarily values below the detection threshold, were imputed with zeros. For comprehensive statistical analyses, we used SPSS (version 27.0, Chicago, IL, USA) along with proprietary software [14]. Principal component analysis (PCA) was conducted using SIMCA software (version 13.0.3; Umetrics). To assess the practical efficacy of our newly developed model for screening purposes, we evaluated its performance using ROC-AUC analysis and a positive predictive value (PPV) that was adjusted based on patient morbidity rates. Specifically, sensitivity and specificity derived from our models were applied to the reported population prevalence of colorectal cancer (124/100,000), gastric cancer (99/100,000), and esophageal cancer (21/100,000), and the adjusted PPV/NPV values were calculated accordingly (Table 2).

**Table 2:** Evaluation of the cancer screening model.

(**A**) Colorectal cancer and healthy participants											
					True State						
					CRC		Healthy		Sum		PPV or NPV
Predicted State	Observed Samples		Positive		489		2		491		
			Negative		411		1778		2189		
			Sum		900		1780		2680		
	Prevalence Correction		Positive		134		112		246		54.5 %
			Negative		113		99641		99754		99.89 %
			Sum		247		99753		100000		
	Sensitivity or Specificity				54.3 %		99.9 %				

(**B**) Gastric adenocarcinoma and healthy participants											
						True State					
						GC		Healthy		Sum	PPV or NPV
Predicted State		Observed Samples		Positive		324		2		326	
				Negative		202		1778		1980	
				Sum		526		1780		2306	
		Prevalence Correction		Positive		61		112		173	35.3 %
				Negative		38		99789		99827	99.96 %
				Sum		99		99901		100000	
		Sensitivity or Specificity				61.6 %		99.9 %			

(**C**) Esophagus cancer and healthy participants											
						True State					
						EC		Healthy		Sum	PPV or NPV
Predicted State		Observed Samples		Positive		84		2		86	
				Negative		40		1778		1818	
				Sum		124		1780		1904	
		Prevalence Correction		Positive		14		112		126	11.1 %
				Negative		7		99867		99874	99.99 %
				Sum		21		99979		100000	
		Sensitivity or Specificity				67.7 %		99.9 %			

CRC, colorectal cancer; GC, gastric cancer; EC, esophageal cancer; PPV, positive predictive rate; NPV, negative predictive rate.

## 3 Results

### 3.1 Comparison of tumor marker levels in samples from gastrointestinal cancer patients and healthy volunteers

**Fig. 1:**
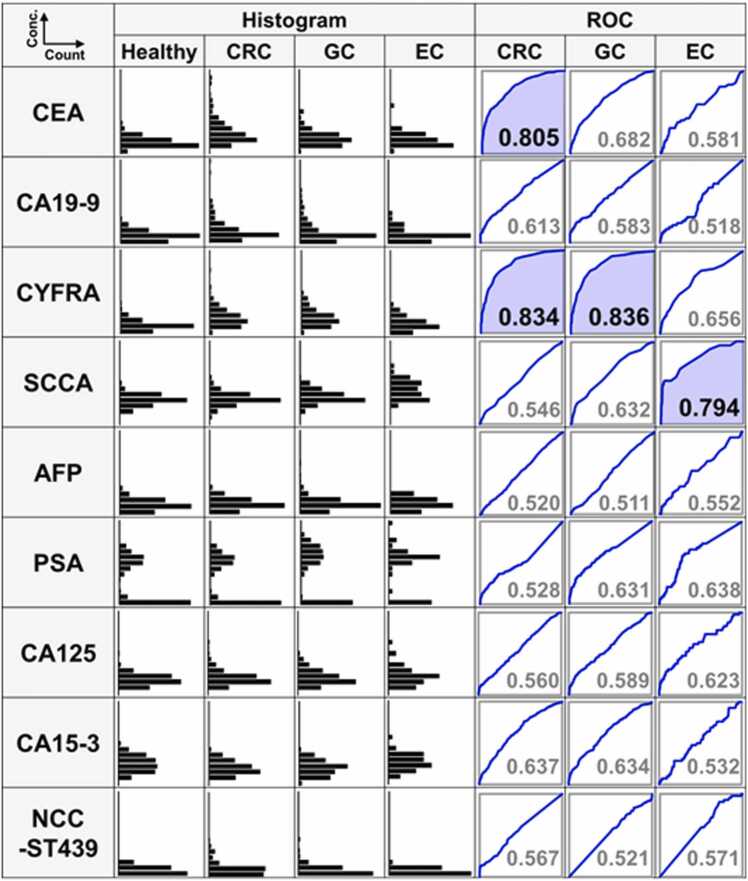
Levels of tumor markers in colorectal cancer (CRC), gastric cancer (GC), esophageal cancer (EC), and healthy volunteer samples. This figure depicts the levels of nine tumor markers, carcinoembryonic antigen (CEA), cytokeratin 19 fragment (CYFRA), alpha-fetoprotein (AFP), carbohydrate antigen 19–9 (CA19–9), cancer antigen 125 (CA125), squamous cell carcinoma antigen (SCCA), nation cancer center-stomach-439 (NCC-ST439), carbohydrate antigen 15–3 (CA15–3), and prostate specific antigen (PSA), in samples from CRC, GC, and EC patients, as well as healthy volunteers. Histograms illustrate the distribution of logarithmically transformed marker levels (log base 10) on the vertical axis against the individual count on the horizontal axis. Additionally, receiver operating characteristic (ROC) curves are shown to compare the diagnostic performance of each marker between the cancer groups (CRC, GC, EC) and healthy group, with the area under the ROC curve (AUC) values indicated. Curves demonstrating AUC values over 0.7 are highlighted with blue shading.

The levels of nine tumor markers in patients with CRC, GC and EC, as well as in healthy volunteers, are presented in histograms (Fig. 1). All values were transformed using a logarithmic scale, with ROC analysis conducted between each cancer group and healthy group. The ROC curves with an AUC value greater than 0.7 are highlighted in blue. Notably, CEA levels were significantly higher in CRC patients compared with the healthy group (AUC = 0.805). CYFRA, which is typically used for diagnosing non-small cell lung cancer [16], showed significant elevation in the CRC (AUC = 0.834) and GC (AUC = 0.836) groups. SCCA, a subfraction of serine protease inhibitors, was specifically elevated in the EC group (AUC= 0.794). CA19–9 showed limited discriminatory ability for CRC (AUC = 0.613). In contrast, other tumor markers—including AFP, CA125, PSA, and CA15–3—did not exhibit significant discriminatory performance for CRC, GC, or EC, as clearly demonstrated in Fig. 1. Their inclusion in the panel was intentional: our aim was to evaluate a broad panel of conventional tumor markers not only for gastrointestinal cancers but also for potential utility in other malignancies. Importantly, because the diagnostic framework relies on machine learning, markers with little or no discriminatory contribution were automatically assigned negligible weights, ensuring that their inclusion did not compromise the performance of the overall model. This approach also enabled the unexpected observation that CYFRA, though primarily used for lung cancer, contributed significantly to discrimination of CRC and GC, highlighting the value of an inclusive panel in discovering unanticipated associations.

### 3.2 Volcano plot analysis, PCA, and heatmap analysis of EGPs in CRC, GC, and EC

**Fig. 2:**
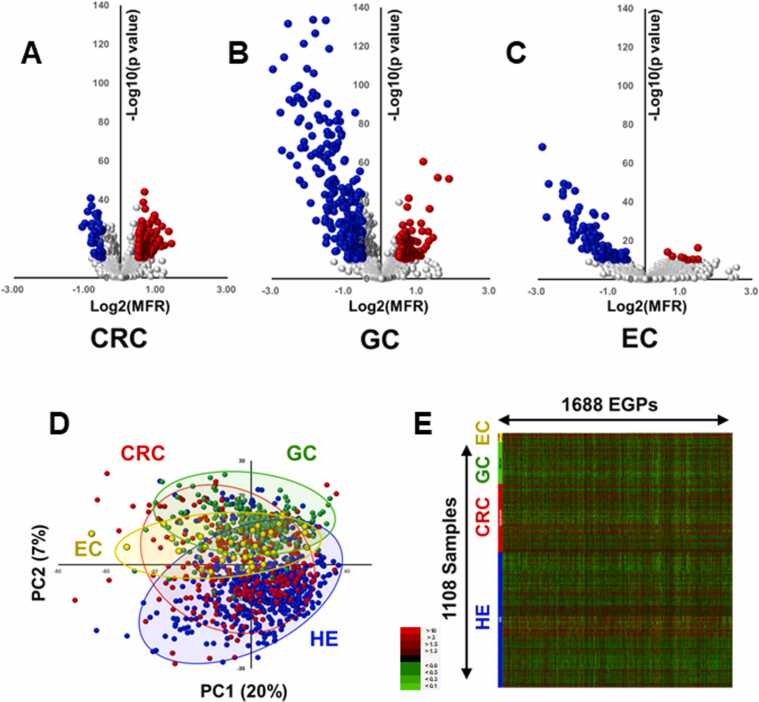
Volcano plot analysis, principal component analysis (PCA), and heatmap analysis of enriched glycopeptides (EGPs) in colorectal cancer (CRC), gastric cancer (GC), and esophageal cancer (EC). A. Volcano plot for comparisons between the CRC and healthy groups. The vertical axis represents the negative logarithm (base 10) of the Student's *t*-test *P*-value and the horizontal axis represents the logarithm (base 2) of the mean-fold ratio (MFR). EGPs with a *P*-value < 1 × 10^−10^ and an MFR > 2^0.5^ are highlighted in red, while those with a *P*-value < 1 × 10^−10^ and an MFR < 2^−0.5^ are highlighted in blue. B. Volcano plot for comparisons between the GC and healthy groups. C. Volcano plot for comparisons between the E**C** and healthy groups. D. PCA score plot showing data for the CRC (red), GC (green), EC (yellow), and healthy (blue) groups. E. Heatmap displaying the levels of 1688 EGPs across 1108 individuals. Upregulated EGPs are shown in red and downregulated EGPs are shown in green.

We extracted 1688 EGPs from the sera of CRC, GC, and EC patients as robust and reliable markers. Volcano plots revealed that the GC samples exhibited a greater number of significantly reduced EGPs compared with the CRC and EC samples, suggesting that GC may have a stronger impact on serum glycans (Fig. 2A–C). PCA demonstrated overlapping distributions between the healthy group and CRC and EC groups. However, the GC group distribution was distinctly separate from those of the other groups, indicating that glycan alterations are more pronounced in GC than in CRC and EC (Fig. 2D). Heatmap analysis also showed a decrease in EGPs in GC compared with the findings in CRC and EC (Fig. 2E). Among the 1688 enriched glycopeptides, a subset including AT271-FSG and MG70-FSG was structurally characterized, revealing that glycosylation site information strongly influenced classification performance. While many detected EGPs remain structurally unassigned, all peaks with retention time and *m/z* have now been provided in Supplementary information to ensure reproducibility.

### 3.3 Identification of novel cancer-specific biomarkers among the EGPs

**Fig. 3:**
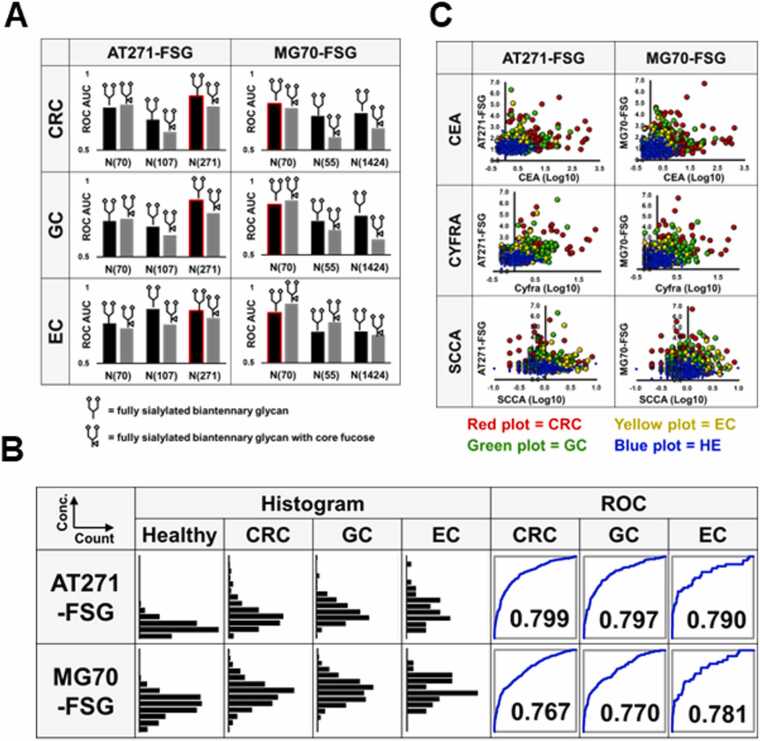
Analysis of α1-antitrypsin (AT)-derived and α2-macroglobulin (MG)-derived glycopeptides. A. Area under the receiver operating characteristic (ROC) curve (AUC) values for glycopeptides from AT and MG, highlighting the glycopeptides used in machine learning with red borders. The numbers following 'N' denote the asparagine sequence position from the N-terminus. B. Histograms displaying the distribution of AT and MG glycopeptide levels across the colorectal cancer (CRC), gastric cancer (GC), esophageal cancer (EC), and healthy (HE) groups. The accompanying ROC curves illustrate the diagnostic accuracy of each glycopeptide, with AUC values indicated for differentiation between the cancer and healthy groups. C. Scatter plots showing the correlation between glycopeptide levels (AT and MG) and tumor markers (carcinoembryonic antigen (CEA), cytokeratin 19 fragment (CYFRA), and squamous cell carcinoma antigen (SCCA)) that are responsive to CRC, GC, or EC. The vertical axes represent the levels of AT or MG, while the horizontal axes show the logarithmically transformed levels of the tumor markers.

In our comprehensive analysis of nearly 10,000 EGPs, we identified promising biomarker candidates that exhibited statistically significant differences between gastrointestinal cancer patients and healthy controls. Specifically, candidates were selected using extremely low Student’s *t*-test *P*-values (< 1 ×10^−10^) and a mean fold-change exceeding 1.5. To control for variability in sample preparation, EGP levels were normalized to transferrin, a protein known for its stable expression across serum samples [17]. Beyond diagnostic significance, candidates were required to demonstrate high reproducibility, defined by intra- and inter-day coefficient of variation (CV) values ≤ 15 %. Ultimately, this led to the discovery of two glycopeptides, α1-antitrypsin (AT) and α2-macroglobulin (MG), which could robustly differentiate CRC, GC, and EC from the healthy groups. Site-specific analysis highlighted the Asn271 site of AT and Asn70 of MG as the most diagnostically informative. Among the glycans detected, only fully sialylated biantennary structures were retained for evaluation due to their quantification robustness. The resulting AT271-FSG and MG70-FSG exhibited AUC values between 0.78 and 0.80 across cancer types, comparable to that of CEA (Fig. 3B). Correlation analysis further demonstrated strong alignment with CYFRA and weak associations with SCCA and CEA (Fig. 3C; Supplementary Table 1), indicating their complementary diagnostic utility.

### 3.4 Combining tumor markers, AT271-FSG and MG70-FSG, and 1688 EPGs significantly enhances the discrimination between cancerous and healthy samples

**Fig. 4:**
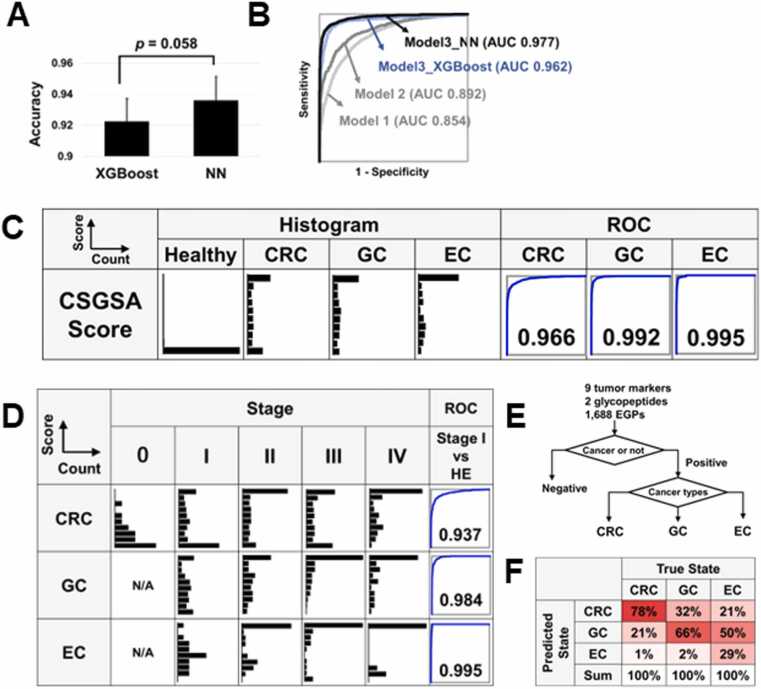
Comprehensive evaluation of the machine learning models and comprehensive serum glycopeptide spectra analysis (CSGSA) score. A. Model comparison: Performance comparison of cancer identification using Model 3 with the XGBoost and Neural Network (NN) algorithms, showcasing accuracy. The classification accuracy was evaluated using the accuracy score from the scikit-learn library of Python library. The accuracy was calculated as the ratio of correctly predicted samples to the total number of samples. B. Receiver operating characteristic (ROC) curves: The ROC curves are displayed for the three different models. C. CSGSA score distribution and performance: Histograms showing the distribution of CSGSA scores across the different groups (colorectal cancer (CRC), gastric cancer (GC), esophageal cancer (EC), and healthy). The adjacent ROC curves evaluate the diagnostic performance of Model 3. D. Staging analysis: Histograms displaying the distribution of CSGSA scores across cancer stages. ROC analysis comparing stage I disease with the healthy group, indicating early detection capability. E. Classification strategy: Outline of the analytical process where individuals are first classified as cancerous or non-cancerous, followed by further classification into specific cancer types (CRC, GC, EC). F. Prediction accuracy: A summary of the cancer type prediction accuracy, presenting the percentage of correct identifications for each cancer type relative to the total predictions made.

To enhance diagnostic accuracy, we constructed a machine learning model that integrates conventional tumor markers with AT271-FSG and MG70-FSG glycopeptides, alongside 1688 EGPs. Three models were developed to evaluate the individual and combined contributions of these markers: Model 1 included only the nine tumor markers; Model 2 added AT271-FSG and MG70-FSG; Model 3 incorporated all markers plus 100 principal components derived from the EGP dataset using PCA. This reduction mitigated overfitting and preserved diagnostic features. The number of PCA components was optimized to maximize the AUC value (Supplementary Table 2). Models were trained on 70 % of the dataset and tested on the remaining 30 %, with 10-fold repetitions to ensure robustness. Results were evaluated using ROC analysis (Supplementary Figure 2). We assessed both XGBoost and neural network (NN) architectures for Model 3. While XGBoost, a gradient boosting method, performs well in classification tasks [18], NNs use multi-layered nodes to capture complex nonlinear relationships [19]. NN outperformed XGBoost in group discrimination (Fig. 4A and Table 2). The AUC scores for distinguishing cancer from healthy samples were 0.854 (Model 1), 0.892 (Model 2), 0.962 (Model 3 using XGBoost), and 0.977 (Model 3 using NN), demonstrating clear improvement over conventional markers (Fig. 4B). We further transformed the predicted values of Model 3 into CSGSA scores (ranging from 0 to 10) using the following equation:

CSGSA score = -log_10_(1 – Model 3 predicted value).

Fig. 4C shows CSGSA score distributions for CRC, GC, and EC. The scores effectively differentiated cancer types from healthy controls, with AUCs of 0.966, 0.992, and 0.995, respectively. A cutoff of 5 was used to classify samples as positive or negative. Sensitivity was 54.3 % for CRC, 61.6 % for GC, and 67.7 % for EC, with specificity exceeding 99.9 % (Table 2). This cutoff was selected to minimize false positives and improve PPV. Importantly, these PPV values (54.5 % for CRC, 35.3 % for GC, and 11.1 % for EC) were not derived from the case-control proportions but were prevalence-adjusted values calculated by applying model sensitivity and specificity to epidemiological prevalence rates (Table 2, “Prevalence Correction”).

### 3.5 The relationship between the CSGSA score and cancer development (stage) and enhancing cancer type classification through NN modeling

Next, the relationship between the CSGSA scores and cancer staging was analyzed. When the CSGSA scores were categorized into five stages, 0, I, II, III, and IV, the CSGSA score was found to be higher in stages II, III, and IV compared with that in stages 0 and I in the three cancer types (Fig. 4D). When comparing stage I patients and the healthy group, the AUC values were 0.94 for CRC, 0.98 for GC, and 0.99 for EC. To enhance the accuracy of classifying gastrointestinal cancer types among positively identified patients, we employed a NN-based classification model (Fig. 4E). ROC analysis of this NN showed remarkable classification accuracies of 78 % for CRC, 66 % for GC, and 29 % for EC (Fig. 4F).

## 4 Discussion

In this study, we propose a novel approach to improving cancer diagnostics by integrating serum EGP profiling with machine learning, alongside conventional tumor markers. Glycosylation changes occur during cancer development and progression [20], [21], and their diagnostic utility remains underexplored. Our goal was to establish a non-invasive, blood-based screening method that reduces patient burden and enhances early detection.

Traditional diagnostics often rely on single biomarkers, which limit sensitivity and specificity. ROC analyses of nine tumor markers (CEA, CA19–9, CYFRA, SCCA, AFP, PSA, CA125, CA15–3, and NCC-ST439) showed inadequate performance when used individually (Fig. 1). These findings collectively indicate that glycosylation patterns, particularly in gastric cancer, are more profoundly altered compared to CRC and EC.

Among the 1688 EGPs analyzed, we focused on glycopeptides derived from AT and MG, identified as cancer-specific (CRC, GC, and EC; Fig. 3A). These peptides showed unique glycosylation at Asn271 (AT271) and Asn70 (MG70), enabling clear distinction from healthy controls. Previous studies reported glycosylation changes in MG [22] and AT [23] during cancer onset. Our study extended this by identifying site-specific alterations in gastrointestinal cancers. ROC analysis showed that AT271-FSG and MG70-FSG outperformed conventional tumor markers (Fig. 3B). Correlation analyses with markers like CEA, CYFRA, and SCCA confirmed that combining AT271-FSG and MG70-FSG improved separation between cancer and non-cancer groups (Fig. 3C), highlighting their potential as novel diagnostic biomarkers.

To build the CSGSA model, we employed machine learning—specifically XGBoost and NN—as conventional analytics could not handle the feature volume. NN outperformed XGBoost (Fig. 4A), likely due to its strength in modeling nonlinear patterns [24], [25]. Among tested models, Model 3, integrating major EGP features, tumor markers (Model 1), and key glycopeptides (Model 2), achieved the best AUC (0.977; Fig. 4B/C), demonstrating improved diagnostic power. Prevalence-adjusted PPVs were 54.5 % (CRC), 35.3 % (GC), and 11.1 % (EC), reflecting EC’s lower incidence. In Japan, EC is less common (27.1/100,000 men) compared to CRC and GC [26]. Despite this, our model exceeded benchmark PPVs (<1 %) [13] and achieved NPV > 99.9 %, supporting its clinical applicability. Compared with liquid biopsies [27] and genomic sequencing [28], CSGSA offers superior accuracy, cost-efficiency, and reproducibility. Notably, the CSGSA score effectively separated cancers from controls (Fig. 4D) and maintained high AUCs in stage I cases (CRC: 0.94, GC: 0.98, EC: 0.99; Fig. 4E), suggesting utility in early detection. It also showed strong prediction for CRC and GC (Fig. 4F/G), which may aid endoscopic triage. Lower EC classification performance likely reflects insufficient sample size for this subtype’s complex biomarker landscape. Importantly, the PPVs were prevalence-adjusted using real-world incidence data, reflecting clinically realistic screening conditions rather than the artificially enriched prevalence of our study cohort.

It should be noted that the CSGSA score is a log-transformed representation of predicted probabilities, designed primarily for visualization and practical cutoff definition. While it facilitates clinical interpretation by expanding intermediate values and providing an intuitive scale, the underlying predicted probabilities are preserved and may also be reported directly.

In addition, although machine learning algorithms such as LightGBM and neural networks are well established, our study demonstrates their novel application in the context of integrating enriched glycopeptide profiling (CSGSA) with conventional tumor markers in gastrointestinal cancers. To our knowledge, this represents the first systematic effort to evaluate this combined approach across colorectal, gastric, and esophageal cancers simultaneously. This study focused on gastrointestinal cancers; however, the same methodology has also been successfully applied in our previous research on ovarian, pancreatic, and liver cancers, underscoring its broader potential across malignancies. This suggests that CSGSA, when combined with machine learning, could serve as a generalizable diagnostic framework extending beyond gastrointestinal cancers.

Beyond the methodological findings, an important consideration is how this approach can be translated into clinical practice. Our implementation strategy is not to provide the algorithm as a stand-alone public webserver, but rather as a centralized laboratory-developed test. In this envisioned model, serum samples would be collected at clinical sites and transferred to a dedicated reference center, where standardized mass spectrometry analysis and AI-based classification would be performed. This centralized approach ensures robust quality control, reproducibility, and stability, which are essential for clinical diagnostics. While we did not include a user-facing webserver in the current work, reproducibility is supported through detailed descriptions of the analysis pipeline in the Methods and supplementary materials. Furthermore, user-friendly reporting tools, including structured diagnostic reports and potentially web-based research interfaces, are under consideration for future development to enhance accessibility for both clinicians and researchers.

In this study, we confirmed the biological plausibility of our approach by structurally characterizing representative EGPs such as AT271-FSG and MG70-FSG, where glycosylation sites showed significant diagnostic contribution. Nevertheless, comprehensive structural annotation of all 1688 detected EGPs remains an ongoing challenge due to the current lack of a complete glycopeptide database. To facilitate reproducibility, we have provided retention time and *m/z* values for all peaks in Supplementary information. Further efforts to fully annotate these glycopeptides will be important to enhance the translational utility of CSGSA.

This study has several limitations. First, it was retrospective and included only patients with confirmed gastrointestinal cancers and healthy controls. Therefore, its use for screening asymptomatic individuals requires validation in prospective studies. Second, there were demographic mismatches between cases and controls: cancer cohorts were generally older and primarily Asian/Caucasian, whereas the control group was younger and included a larger proportion of African American and Hispanic participants. These imbalances may introduce confounding and spectrum bias. Although our models were trained on mixed datasets and the reported PPVs were prevalence-adjusted to reflect real-world incidence (Table 2), this limitation cannot be fully excluded. Validation in age- and ancestry-matched prospective cohorts will be required to confirm the generalizability of our findings. Third, while mass spectrometry enables comprehensive EGP analysis, it requires specialized equipment. Simpler, scalable diagnostic methods are needed. Though endoscopy remains the gold standard, its cost and complexity hinder widespread use, particularly in underserved areas. Mass spectrometry-based EGP analysis may become more accessible with scale and automation, potentially lowering costs for broad implementation. Forth, the dataset for machine learning model training was relatively small, limiting model optimization, especially for EC, which showed lower predictive accuracy. Larger cohorts or additional biomarkers are needed. Fifth, the study population was primarily Asian and Caucasian, leaving applicability in other ethnic groups uncertain. As suggested in Supplementary Figure 3, ethnicity may influence CSGSA scores, indicating a need for international validation. To assess the potential impact of racial differences, we performed an additional analysis using only the Asian population, which represented the majority of our cohort. Following the same neural network–based approach described in Supplementary Figure 2, we trained and evaluated the model using Asian samples exclusively. As shown in Supplementary Figure 4, the ROC-AUC for all cancers decreased by only 0.012 compared with the mixed population including Caucasians, African Americans, and Hispanics, and the largest difference among individual cancer types was 0.016 for gastric cancer. These minor differences indicate that the proposed method is largely robust to ethnic variability.

Sixth, although we minimized overfitting by randomly dividing the dataset into independent training and test sets (70:30), we did not perform more rigorous internal validation approaches such as nested cross-validation or leave-one-site-out analysis. The latter would specifically test robustness across different contributing institutions. This limitation may lead to some degree of spectrum bias, and therefore validation in independent, age- and site-matched cohorts will be required in future studies. Finally, the model focused solely on gastrointestinal cancers. Its utility for other cancer types—such as lung, pancreatic, or breast cancer—remains unknown. Future research should evaluate CSGSA performance in diverse cancer types.

## 5 Conclusions

Combining glycopeptide profiling with NN-based machine learning models successfully discriminated CRC, GC, and EC from healthy controls. This approach achieved remarkable AUC values of 0.966, 0.992, and 0.995, respectively, as well as superior PPVs of 54.5 %, 35.3 %, and 11.1 %, respectively, outperforming the existing tumor markers. Our findings present a novel diagnostic framework leveraging glycosylated peptides, marking a significant step toward the future of cancer diagnosis. With further research and technological advancements, CSGSA is anticipated to become a new clinical standard for gastrointestinal cancer diagnostics.

## List of abbreviations

CRC: colorectal cancer

GC: gastric cancer

EC: esophageal cancer

AFP: alpha-fetoprotein

PSA: prostate specific antigen

CEA: carcinoembryonic antigen

CA125: cancer antigen 125

CA15–3: carbohydrate antigen 15–3

CA19–9: carbohydrate antigen 19–9

CYFRA: cytokeratin 19 fragment

SCCA: squamous cell carcinoma antigen

NCC-ST439: nation cancer center-stomach-439

CSGSA: comprehensive serum glycopeptide spectra analysis

LC-MS: liquid chromatography-mass spectrometry

QC: quality control

PPV: positive predictive value

## Authors' contributions

• **Yuichi Hisamatsu (YH)** and **Kazuhiro Tanabe (KT)** conceptualized and designed this study.
• **Kazuhiro Tanabe (KT)** reviewed previous studies and formulated the hypothesis.
• **Yuichi Hisamatsu (YH)**, **Kensuke Kudo (KK)**, and **Tetsuya Kusumoto (TK)** recruited participants, obtained informed consent, prepared specimens, collected clinical data, and created case report forms.
• **Hirofumi Hasuda (HH)** and **Yoshihisa Sakaguchi (YS)** contributed to data curation and resource management.
• **Eiji Kusumoto (EK)**, **Hideo Uehara (HU)**, **Rintaro Yoshida (RY)**, and **Mitsuhiko Ota (MO)** contributed to resource provision, data acquisition, and manuscript review.
• **Kazuhiro Tanabe (KT)** and **Mikio Mikami (MM)** developed the machine learning model using Python.
• **Chihiro Hayashi (CH)** conducted the glycopeptide analysis using LC-MS, identified glycopeptide structures, and validated the methodology.
• **Kazuhiro Tanabe (KT)** wrote the original draft of the manuscript.
• **Yuichi Hisamatsu (YH)** and **Tetsuya Kusumoto (TK)** supervised the study.
• All authors reviewed and/or edited the final manuscript and approved the decision to submit it.

## CRediT authorship contribution statement

**Hideo Uehara:** Resources. **Rintaro Yoshida:** Resources. **Mitsuhiko Ota:** Resources. **Yoshihisa Sakaguchi:** Resources, Data curation. **Hirofumi Hasuda:** Resources, Data curation. **kusumoto Eiji:** Resources. **Kensuke Kudo:** Writing – review & editing, Investigation, Data curation. **Chihiro Hayashi:** Writing – review & editing, Validation, Methodology, Investigation. **Mikio Mikami:** Writing – review & editing, Software, Formal analysis. **Tetsuya Kusumoto:** Writing – review & editing, Supervision, Investigation, Data curation. **Yuichi Hisamatsu:** Writing – review & editing, Writing – original draft, Supervision, Project administration, Methodology, Investigation, Conceptualization. **Kazuhiro Tanabe:** Writing – review & editing, Writing – original draft, Software, Methodology, Formal analysis, Conceptualization.

## Ethics approval and consent to participate

The Institutional Review Boards of National Hospital Organization Kyushu Medical Center (IRB registration number: 17C299; December 20, 2017) and LSI Medience Corporation (IRB registration number: MS/Shimura 17–19; January 22, 2018) approved the use of patient clinical information and serum samples. All research methods and procedures were conducted strictly according to the ethical principles outlined in the Declaration of Helsinki, as well as in full compliance with relevant institutional, national, and international guidelines. We obtained written informed consent from all patients and volunteers.

## Declaration of Generative AI and AI-assisted technologies in the writing process

During the preparation of this work, the authors used ChatGPT to improve English expression and grammar. After applying these edits, the authors reviewed and revised the manuscript as needed and take full responsibility for the content of the published article. In addition, the manuscript underwent professional English editing by J. Iacona, Ph.D., from Edanz (http://jp.edanz.com/ac).

## Funding

This research did not receive any specific grant from funding agencies in the public, commercial, or not-for-profit sectors. It was solely funded by internal resources of LSI Medience Corporation.

## Declaration of Competing Interest

KT and CH are employed by LSI Medience Corporation, which can provide cancer screening. LSI Medience Corporation applied for a patent related to this research in Japan (Toku-gan 2023–105968). The remaining authors declare that they have no competing interests.

## Data Availability

The levels of nine tumor markers, AT271-FSG and MG glycopeptides, and EGPs, along with anonymized case information, are available for disclosure. The data will be accessible after publication. Researchers seeking raw data should contact the corresponding author via email. Access requires approval from each institution's ethics committee and a collaborative agreement with the corresponding author.

## Footnotes

Appendix A Supplementary data associated with this article can be found in the online version at doi:10.1016/j.csbj.2025.10.067.
