# Supplementary material for: Comprehensive serum glycopeptide spectrum analysis with machine learning for non-invasive early detection of gastrointestinal cancers
Source: Comput Struct Biotechnol J. 2025 Nov 1;27:4792–801. doi: 10.1016/j.csbj.2025.10.067 (PMC12636384; doi:10.1016/j.csbj.2025.10.067)
Supplement: Supplementary file 5 — Supplementary material [file mmc5.docx]

**Supplementary Data**


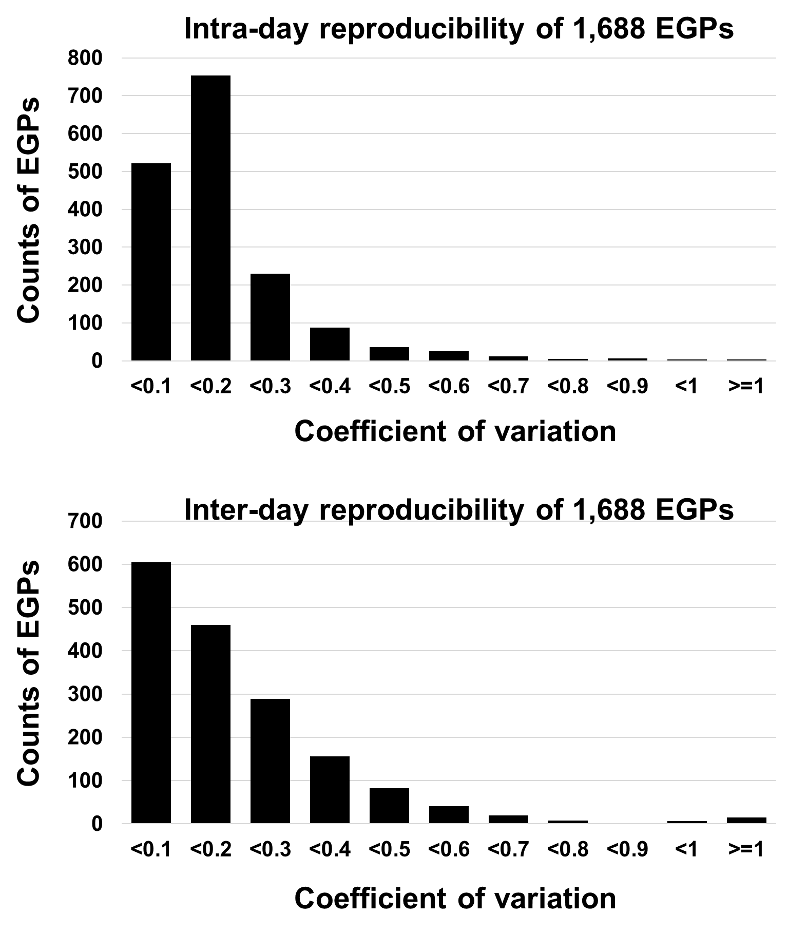


**Supplementary Figure 1.** **Intra-day and inter-day reproducibility of 1,688 enriched glycopeptides (EGPs).**

Each EGP was measured five times within the same day (including preparation and MS measurement errors). The coefficient of variation (CV) was calculated for each EGP and presented as a histogram. For the inter-day reproducibility, measurements were taken five times per day, with the average of these values then obtained. This process was repeated over three days. The CV values of the mean values (n = 3) are shown in the histogram.

**Supplementary Figure 2. Machine learning model development and evaluation strategy.**

This figure outlines the development and evaluation of three distinct machine learning models to assess the impact of different biomarker sets on cancer diagnosis accuracy. Model 1 incorporates nine conventional serum tumor markers, Model 2 extends this to include glycopeptides associated with α1-antitrypsin (AT) and α2-macroglobulin (MG), and Model 3 integrates these components with 100 key glycan features derived from a principal component analysis (PCA) of 1,688 enriched glycopeptides (EGPs). Each model was trained using a randomly selected training set containing 70% of the samples, with the remaining 30% serving as the test set. The evaluation process was repeated 10 times to validate the consistency. The results were collectively analyzed through receiver operating characteristic (ROC) curve analysis to measure the performance across various configurations.

**Supplementary Figure 3. Distribution of the Comprehensive Serum Glycopeptide Spectra Analysis (CSGSA) scores by tumor differentiation and ethnicity, as well as key feature contributions in Model 3.
A.** Histograms of the CSGSA scores for the colorectal cancer (CRC) and gastric cancer (GC) groups, categorized by differentiation degree: well-differentiated, moderately differentiated, and poorly differentiated. An insufficient sample size prevented analysis of the esophageal cancer (EC) group. **B.** Relationships between the CSGSA scores and ethnicity. Histograms depicting the CSGSA scores across healthy individuals and patients with CRC, GC, or EC in Asian and Caucasian populations. The African American and Hispanic groups are represented in histograms for healthy individuals only. **C.** Contributors to Model 3 identified through XGBoost F Scores, visualizing the contribution of each factor to the model. PC1 through PC8 represent the first to eighth principal components, respectively, derived from a principal component analysis of the 1,688 enriched glycopeptides (EGPs). These components were used to develop Model 3.

**Supplementary Text (with Supplementary Figure 3)**

**Key contributors to model efficacy**

Identifying the key contributors to the construction of the neural network model is crucial. However, the neural network framework significantly limits the visibility into which explanatory variables influence the model's outcomes. In contrast, XGBoost allows for the identification of such contributions. Therefore, we used this to estimate the contributing factors, although it was not direct. In XGBoost, the 'F Score' indicates the frequency with which a feature is used to split the data across all trees in the model. A higher F Score suggests that the feature played a more significant role in creating a model, thus being crucial for the model’s decision-making process. Our results showed that CEA and CYFRA had strong contributions (**Supplementary Figure 3C**), corroborated by their high AUC values (Fig. 1). Interestingly, CA15-3 also made a significant impact on the model, notably from its decreased levels in the CRC and GC groups. Among the PCA-derived features, PC2 was particularly influential, corroborated by the clear separation between the cancer and healthy groups in PC2, as shown in Figure 2D.

Our study also highlights the potential of integrating advanced machine learning techniques with molecular diagnostics. Future iterations of this research could leverage newer machine learning models to further refine the sensitivity and specificity of the diagnostic process. Additionally, continuous updates to the glycopeptide databases and improvements in mass spectrometry sensitivity could provide even more robust datasets for analysis.


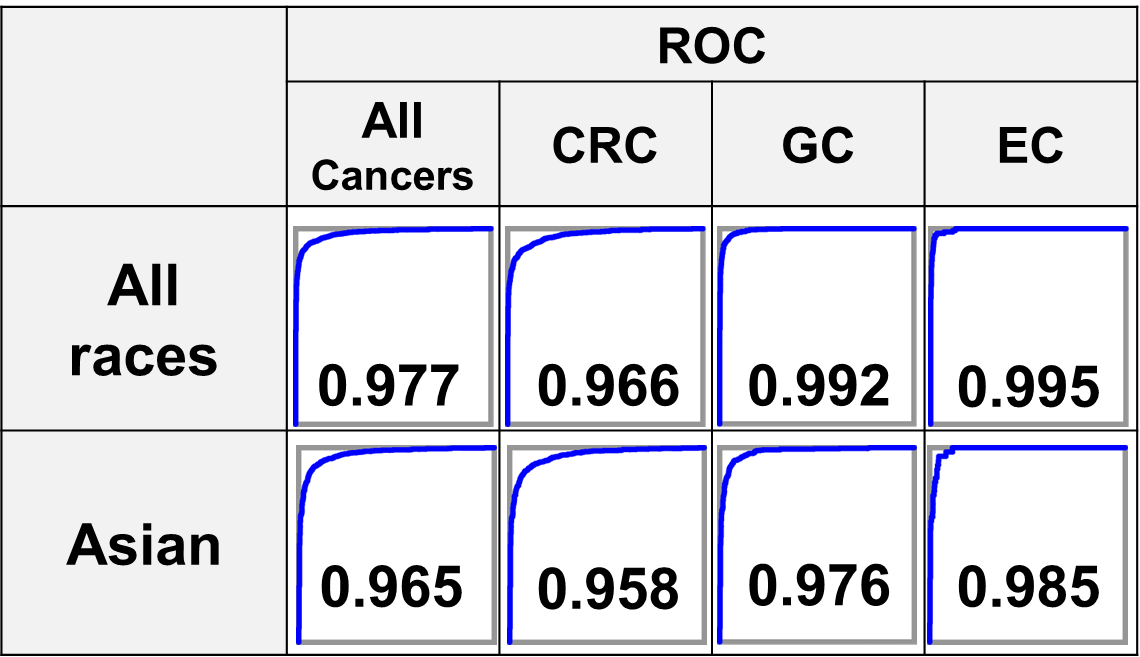


**Supplementary Figure 4. Receiver operating characteristic (ROC) analysis comparing model performance between all-race and Asian-only cohorts.**
ROC curves illustrate the diagnostic performance of the neural network–based CSGSA model when trained and tested on (top) the full multiethnic dataset and (bottom) an Asian-only subset.
The area under the curve (AUC) values were 0.977 (all cancers), 0.966 (CRC), 0.992 (GC), and 0.995 (EC) in the full cohort, and 0.965, 0.958, 0.976, and 0.985 in the Asian-only subset, respectively.
The minimal AUC deviations (ΔAUC ≤ 0.016) indicate that the model’s diagnostic accuracy was largely preserved across different ethnic compositions, demonstrating that the CSGSA framework is robust and minimally affected by racial or ancestry-related variability.

**Supplementary Table 1. ROC AUC and 95%CI**

| Item | Class | AUC | 95%CI |
| --- | --- | --- | --- |
| CSGSA | CRC | 0.966 | (95% CI: 0.959–0.973) |
|  | GC | 0.992 | (95% CI: 0.989–0.994) |
|  | EC | 0.995 | (95% CI: 0.992–0.998) |
| CSGSA Stage 1 | CRC | 0.937 | (95% CI: 0.917–0.956) |
|  | GC | 0.984 | (95% CI: 0.978–0.989) |
|  | EC | 0.995 | (95% CI: 0.991–0.999) |
| AT271-FSG | CRC | 0.799 | (95% CI: 0.767–0.830) |
|  | GC | 0.797 | (95% CI: 0.757–0.835) |
|  | EC | 0.790 | (95% CI: 0.703–0.871) |
| MG70-FSG | CRC | 0.767 | (95% CI: 0.733–0.799) |
|  | GC | 0.770 | (95% CI: 0.728–0.809) |
|  | EC | 0.781 | (95% CI: 0.698–0.858) |
| CEA | CRC | 0.805 | (95% CI: 0.775–0.835) |
|  | GC | 0.682 | (95% CI: 0.637–0.727) |
|  | EC | 0.581 | (95% CI: 0.480–0.680) |
| CA19-9 | CRC | 0.613 | (95% CI: 0.572–0.653) |
|  | GC | 0.583 | (95% CI: 0.533–0.633) |
|  | EC | 0.518 | (95% CI: 0.426–0.609) |
| CYFRA | CRC | 0.834 | (95% CI: 0.806–0.861) |
|  | GC | 0.836 | (95% CI: 0.801–0.868) |
|  | EC | 0.656 | (95% CI: 0.561–0.747) |
| SCCA | CRC | 0.546 | (95% CI: 0.506–0.584) |
|  | GC | 0.632 | (95% CI: 0.585–0.679) |
|  | EC | 0.794 | (95% CI: 0.713–0.868) |
| AFP | CRC | 0.520 | (95% CI: 0.480–0.559) |
|  | GC | 0.511 | (95% CI: 0.462–0.556) |
|  | EC | 0.552 | (95% CI: 0.455–0.644) |
| PSA | CRC | 0.528 | (95% CI: 0.487–0.568) |
|  | GC | 0.631 | (95% CI: 0.581–0.680) |
|  | EC | 0.638 | (95% CI: 0.550–0.723) |
| CA125 | CRC | 0.560 | (95% CI: 0.521–0.601) |
|  | GC | 0.589 | (95% CI: 0.539–0.638) |
|  | EC | 0.623 | (95% CI: 0.527–0.720) |
| CA15-3 | CRC | 0.637 | (95% CI: 0.600–0.676) |
|  | GC | 0.634 | (95% CI: 0.587–0.678) |
|  | EC | 0.532 | (95% CI: 0.441–0.622) |
| NCC-ST439 | CRC | 0.567 | (95% CI: 0.528–0.606) |
|  | GC | 0.521 | (95% CI: 0.476–0.566) |
|  | EC | 0.571 | (95% CI: 0.496–0.642) |

**Supplementary Table 2. Optimization of Principal Component Count for Model 3 Based on ROC-AUC Performance**

| Number of PCA components | 100 | 200 | 50 | Non |
| --- | --- | --- | --- | --- |
| ROC-AUC | 0.936 | 0.928 | 0.920 | 0.924 |
